# Can Fractional Flow Reserve-Computed Tomography (FFR-CT) Overestimate the Severity of Coronary Stenoses in the Presence of High Calcium Burden?

**DOI:** 10.7759/cureus.94826

**Published:** 2025-10-17

**Authors:** Carly A Robinson, Jade Tso, Lemuel Rivera, Ahmadreza Ghasemiesfe, Saul Schaefer

**Affiliations:** 1 Medical School, UC Davis School of Medicine, Sacramento, USA; 2 Internal Medicine, UC Davis Health, Sacramento, USA; 3 Radiology, Division of Cardiothoracic Imaging, UC Davis Health, Sacramento, USA; 4 Internal Medicine, Division of Cardiovascular Medicine, UC Davis Health, Sacramento, USA

**Keywords:** cardiac catheterization, case report, computed tomography, coronary artery calcium, coronary artery disease, fractional flow reserve-computed tomography (ffr-ct)

## Abstract

Non-invasive diagnostic methods such as coronary artery calcium (CAC) scoring, computed tomography angiography (CTA), and fractional flow reserve-computed tomography (FFR-CT) are useful to predict the presence of coronary artery stenosis, but may provide false positive results as well. This case describes a man in his mid-60s with significant cardiac history who presented to the emergency department with chest pain. Extensive workup included CTA, which demonstrated severe coronary artery calcification and abnormal FFR-CT suggestive of severe three-vessel disease. Urgent invasive coronary angiography was performed due to his high likelihood of flow-limiting stenosis; however, it revealed only mild-to-moderate non-obstructive (<50%) stenoses. This report highlights the potential of CAC, CTA, and FFR-CT to overestimate the severity of coronary artery disease.

## Introduction

Coronary artery disease (CAD) is a common cause of morbidity and mortality in industrialized nations [1]. Coronary artery calcium (CAC) scores are widely recognized as a helpful predictor of cardiovascular event risk in asymptomatic individuals. First described in 1990, the amount of calcium noted on coronary computed tomography (CT) is quantified into a CAC score, thereby providing a non-invasive manner to risk-stratify patients [2]. Further assessment of coronary stenoses can be evaluated through computed tomography angiography (CTA), which provides an anatomic image of the coronary arteries [3,4]. CTA can be coupled with CT-derived fractional flow reserve (FFR-CT), which generates a 3D model of the heart along with predicted flow through the coronary arteries [3]. While CAC scores, CTA, and FFR-CT are useful as non-invasive methods for CAD prediction, they may at times provide false positive results of significant stenosis [5-7]. 

This case describes a patient with a significant cardiac history, including hypertension, congestive heart failure, and atrial fibrillation, who was found to have a markedly elevated CAC score and abnormal CTA and FFR-CT suggestive of severe three-vessel disease, while invasive coronary angiography revealed only non-obstructive stenoses of <50%. This report highlights the potential of FFR-CT to overestimate CAD severity, specifically in the setting of elevated CAC. This case may be useful for clinicians involved in the workup of CAD, especially when concerning patients whose CAC score is elevated. 

## Case presentation

A man in his mid-60s with a significant cardiovascular history, including recent subacute type B aortic dissection with surgical repair and on anticoagulation for prior massive pulmonary embolism, presented to the emergency department (ED) with recurrent severe chest, back, and abdominal pain and diaphoresis for one week. He had been referred to the ED with these symptoms from a vascular surgery clinic visit and was then admitted to the inpatient vascular surgery service. 

Initial blood pressure in the ED was 130/84. There was no chest wall tenderness and no audible murmurs. He had mild wheezing bilaterally and minimal suprapubic tenderness. Cardiology was consulted to evaluate this patient’s chest pain in the setting of known systemic vascular disease. Aside from his recent pulmonary embolism one month prior to admission, other medical problems included heart failure with preserved ejection fraction (HFpEF) of 60%, paroxysmal atrial fibrillation, hypertension, ischemic stroke, squamous cell carcinoma of the head and neck status post chemotherapy and radiation, and May-Thurner syndrome. 

Given his presentation, possible diagnoses of acute coronary syndrome due to coronary artery disease, recurrent pulmonary embolism due to cancer-related prothrombotic state, acute aortic syndrome given his history of prior aortic dissection, and heart failure exacerbation given his history of HFpEF were all considered. Initial electrocardiogram revealed a normal sinus rhythm without acute ischemic changes. Chest X-ray showed cardiomegaly with mild vascular congestion, but no significant pulmonary edema. There was mild troponin T elevation at 38 ng/L (normal < 19 ng/L) 10 hours after admission and NT-proBNP (N-terminal pro-B-type natriuretic peptide) elevation at 2,701 pg/mL (normal < 200 pg/mL). Echocardiogram during hospitalization revealed an ejection fraction of 55%, consistent with his prior diagnosis of HFpEF. CT angiography (CTA) of the chest did not reveal pulmonary artery filling defects nor leakage of his aortic graft repair. His HEART score was 4, warranting further risk stratification [8]. Positron emission tomography (PET) nuclear stress testing with regadenoson and rubidium-82 on the day following the echocardiogram showed normal left ventricular myocardial perfusion but global hypokinesis at both rest and stress, with decreased left ventricular ejection fraction of 40% at rest and 47% at stress. No regional perfusion defects were noted at either rest or stress imaging. Coronary flow reserve, a measure of small vessel disease, could not be determined from this exam due to technical reasons. 

**Figure 1:**
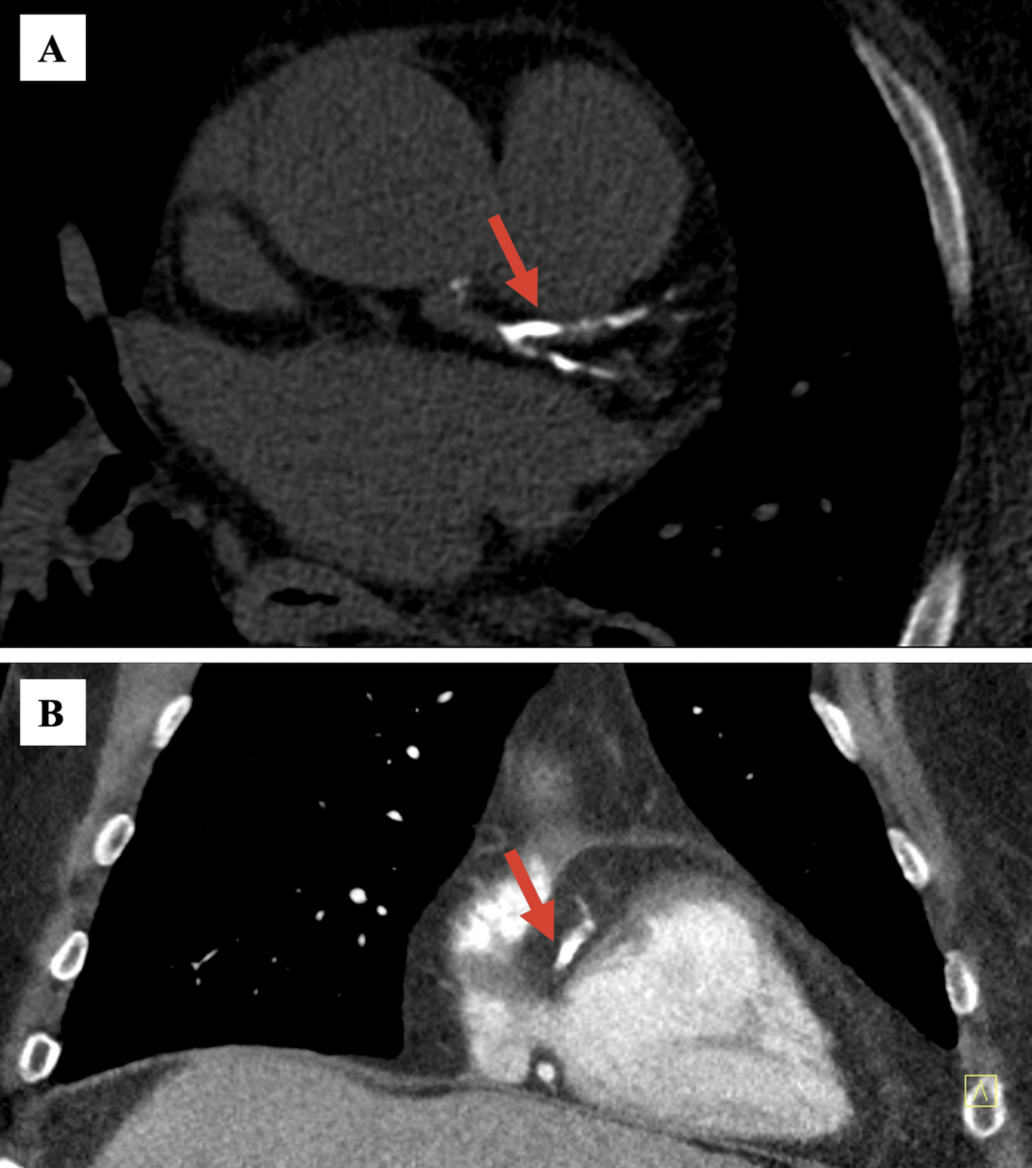
Computed tomography angiogram (CTA) results. Contrast-enhanced CTA images of severe (A) left anterior descending artery calcifications in the transverse plane and (B) right coronary artery calcifications in the coronal plane (red arrows), prompting suspicion for flow-limiting stenoses.

**Figure 2:**
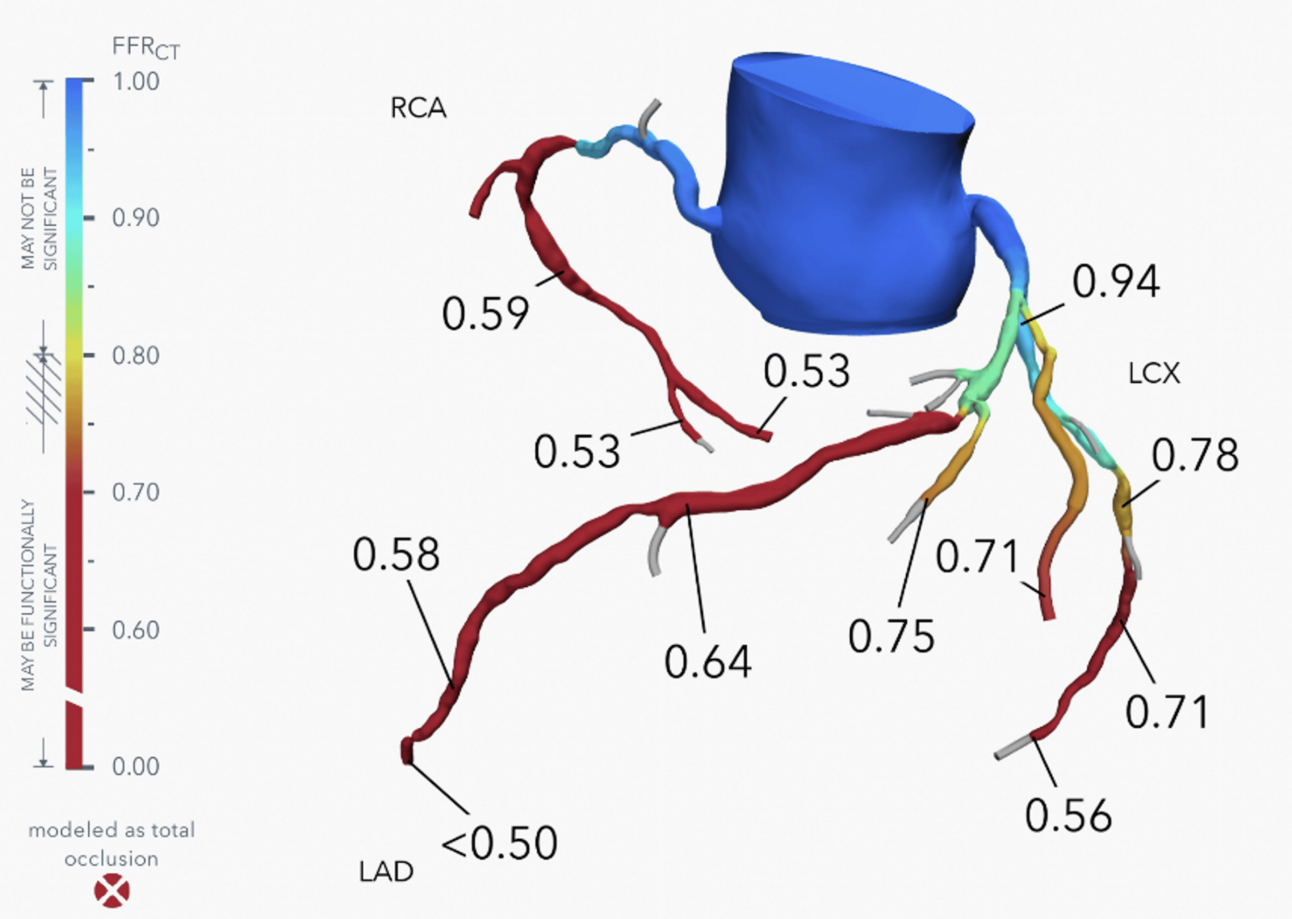
Computed tomography-derived fractional flow reserve (FFR-CT) report. FFR-CT values for left anterior descending artery 0.64, right coronary artery 0.59, and left circumflex artery 0.71, demonstrating a high likelihood of flow-limiting stenosis.

**Figure 3:**
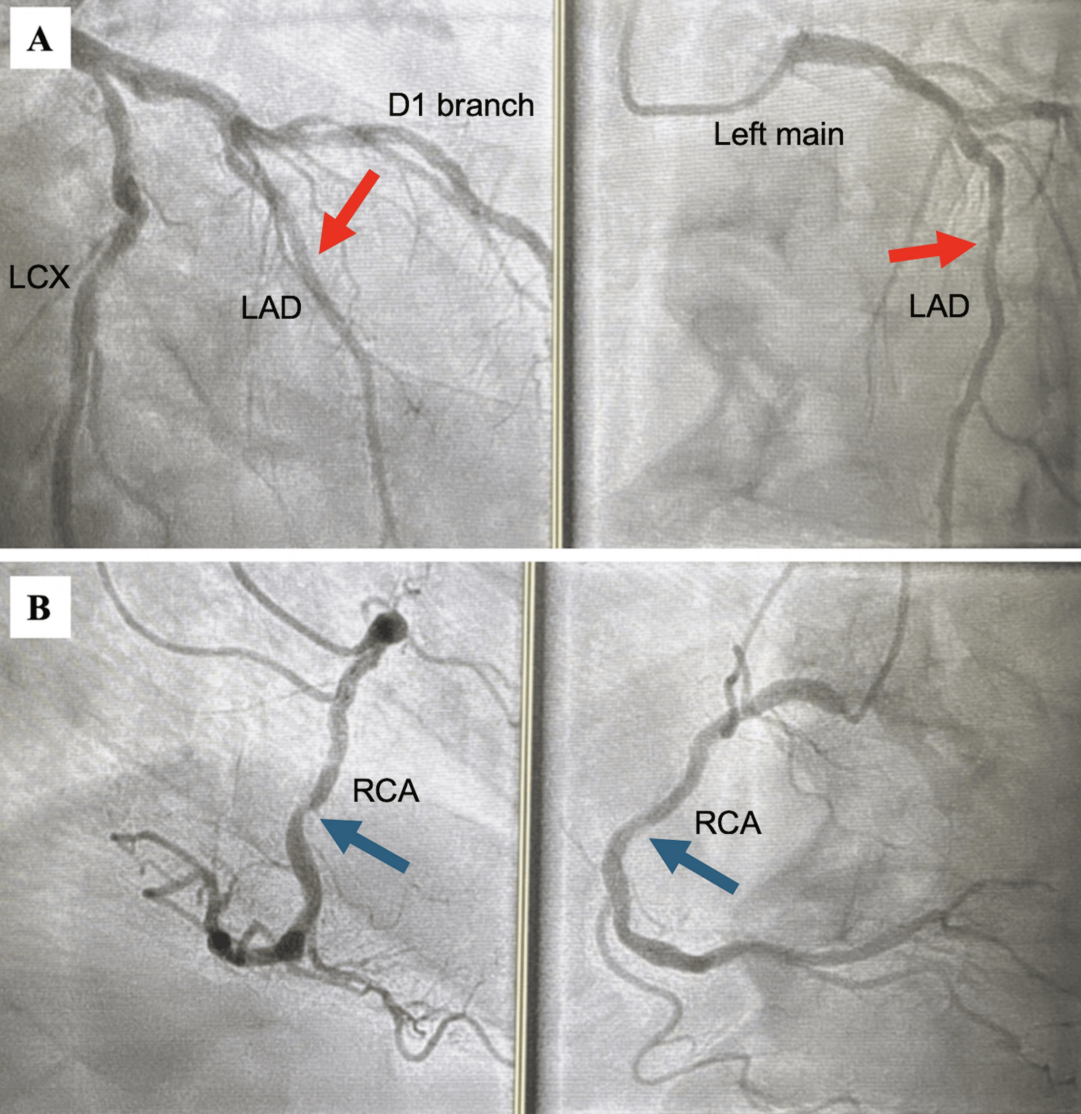
Cardiac catheterization results. Cardiac catheterization images demonstrating mild-to-moderate coronary artery disease with (A) 39% stenosis in the left anterior descending artery (red arrows) and (B) 47% stenosis in the right coronary artery (blue arrows) as determined by QCA.

Given the history and clinical findings of systemic atherosclerotic disease, a diagnosis of balanced ischemia due to three-vessel CAD was considered. Follow-up coronary CTA demonstrated severe coronary artery calcifications (Figure 1) with a total CAC score of 2071 Agatston units. FFR-CT analysis was consistent with severe multivessel CAD with likely intermediate moderate-to-severe stenosis involving the left anterior descending artery (LAD; FFR-CT 0.64), right coronary artery (RCA; FFR-CT 0.59), and left circumflex artery (LCX; FFR-CT 0.71) (Figure 2). Normal FFR-CT values are >0.80 [3]. As FFR-CT values were associated with a high likelihood of flow-limiting stenosis, urgent invasive coronary angiography was performed. However, this study only demonstrated mild-to-moderate CAD as determined by visual assessment by two experienced operators and confirmed by QCA (quantitative coronary angiography) (Figure 3). No further invasive measurements were performed (e.g., FFR, optical coherence tomography, intravascular ultrasound). Since it is known that these invasive measurements increase the accuracy of coronary angiography [9], it is possible that the visual assessment in this instance was inaccurate. However, the negative PET stress test supported the absence of severe flow-limiting coronary stenoses. 

The patient was treated with apixaban 5 mg twice daily, aspirin 81 mg daily, atorvastatin 80 mg daily at bedtime, carvedilol 12.5 mg twice daily, and amlodipine 10 mg daily. He was discharged in stable condition after five days of admission and was followed by cardiology outpatient with no further episodes of chest pain. 

## Discussion

Elevated CAC scores are linked to a higher risk of major adverse cardiovascular events and mortality in a dose-dependent manner [10]. The relative risk of major cardiovascular events compared to patients with CAC score of zero has been reported as 4.3 for a CAC score of 100-400, 7.2 for a CAC score of 401-999, and 10.8 for a CAC score >1000 [10]. Furthermore, Rijlaarsdam-Hermsen et al. found over an eight-year period that patients with a CAC score <100 had a 95% survival rate, patients with a CAC score of 100-400 had a 90% survival, and patients with a CAC score >400 had an 82% survival rate [11]. 

Despite the recognized utility of CAC scores for defining prognosis, there are limitations in assessing the severity of flow-limiting coronary stenoses. Specifically, the presence of CAC has not been shown to anatomically define the location or severity of coronary artery stenoses. A meta-analysis found that a non-zero CAC score had a 98% sensitivity, 40% specificity, 93% negative predictive value, and 68% positive predictive value for detection of >50% stenosis on cardiac catheterization [5]. 

Prior studies indicate that elevated CAC scores and/or certain calcium characteristics, such as calcium length and volume, can compromise the diagnostic performance of CTA [4,6,12]. Per Diederichsen et al, the specificity of CTA in identifying vessels with >50% stenosis significantly decreases with CAC >400 (specificity 17%) versus CAC <400 (specificity 91%) [6]. A meta-analysis of 19 studies supported these findings, showing a reduced specificity of 42% for patients in the highest CAC score category of >400 [12]. These studies suggest that patients with markedly high CAC scores, such as our patient with CAC >1000, are at risk of CTA providing false positive stenosis findings. As a result, Abdulla et al. recommended that future studies examine the utility of CTA with CAC scores above 400 [12]. 

To increase CTA accuracy and assess the functional impact of coronary stenoses, FFR can be derived from CTA. FFR-CT creates a 3D model of the heart with subsequent analysis of predicted flow through the coronary arteries. FFR-CT values below 0.8 are abnormal and can be used to define the presence of coronary artery stenosis >50%. The NXT trial found that FFR-CT was a highly accurate and cost-effective predictor of stenosis >50% compared to invasive angiography [3], and a substudy reported that FFR-CT performance was not significantly impacted between low/mid CAC values of 0-415 and high CAC values of 416-3,599 [13]. In addition to assessing the severity of discrete epicardial coronary artery stenoses, FFR-CT can also be abnormal in the setting of diffuse atherosclerotic disease or significant small-vessel disease [14]. 

However, as anatomic CTA imaging is impacted by elevated CAC, FFR-CT can be negatively influenced as well. Tesche et al. later found that while FFR-CT was superior to anatomic coronary CTA alone across CAC scores, FFR-CT had significantly decreased diagnostic performance when evaluating vessels with CAC scores>400, compared to CAC >0 and <400 [7]. This study also noted a poor correlation of FFR-CT versus invasive FFR findings at CAC >400 [7], further highlighting the limitations of noninvasive diagnostic tests such as CTA and FFR-CT. 

## Conclusions

Non-invasive methods, including CAC, CTA, and FFR-CT, effectively predict coronary artery stenosis, but in some instances may overestimate CAD burden. We present a case of a patient with chest pain who had a markedly elevated CAC score and abnormal FFR-CT suggestive of severe three-vessel disease. However, cardiac catheterization demonstrated only mild-to-moderate non-obstructive CAD. This case highlights the potential of incorrect diagnosis of hemodynamically significant coronary artery stenoses by FFR-CT in the setting of elevated CAC. Clinicians should therefore be aware of the limitations of FFR-CT in the setting of elevated CAC and utilize adjunctive measures to assess the burden of atherosclerosis. This limitation is likely more significant as the CAC increases without a known threshold.
